# Conserved Active-Site Residues Associated with OAS Enzyme Activity and Ubiquitin-Like Domains Are Not Required for the Antiviral Activity of goOASL Protein against Avian Tembusu Virus

**DOI:** 10.3390/v10070371

**Published:** 2018-07-15

**Authors:** Shun Chen, Chao Yang, Jinyue Zhang, Zhen Wu, Mingshu Wang, Renyong Jia, Dekang Zhu, Mafeng Liu, Qiao Yang, Ying Wu, Xinxin Zhao, Shaqiu Zhang, Yunya Liu, Ling Zhang, Yanling Yu, Yu You, Anchun Cheng

**Affiliations:** 1 Institute of Preventive Veterinary Medicine, College of Veterinary Medicine, Sichuan Agricultural University, Chengdu 611130, China; 15108224618@163.com (C.Y.); zhangjinyue@sicau.cn (J.Z.); wu_zhen666@outlook.com (Z.W.); mshwang@163.com (M.W.); cqrc_jry@163.com (R.J.); liumafengra@163.com (M.L.); yangqiao721521@sina.com (Q.Y.); yingzi_no1@126.com (Y.W.); xxinzhao@sicau.edu.cn (X.Z.); shaqiu86@hotmail.com (S.Z.); yunnyaaliu@163.com (Y.L.); zl97451@126.com (L.Z.); yanling3525@163.com (Y.Y.); youyu3508@gmail.com (Y.Y.); 2 Research Center of Avian Disease, College of Veterinary Medicine, Sichuan Agricultural University, Chengdu 611130, China; zdk24@sicau.edu.cn; 3 Key Laboratory of Animal Disease and Human Health of Sichuan Province, Sichuan Agricultural University, Chengdu 611130, China

**Keywords:** goose, 2′-5′-oligoadenylate synthetase-like, duck-origin Tembusu virus, antiviral activity, 2′-5′-oligoadenylate synthetase enzyme activity, ubiquitin-like domains

## Abstract

Interferon (IFN)-induced 2′-5′-oligoadenylate synthetase (OAS) proteins exhibit an extensive and efficient antiviral effect against flavivirus infection in mammals and birds. Only the 2′-5′-oligoadenylate synthetase-like (*OASL*) gene has been identified thus far in birds, except for ostrich, which has both *OAS1* and *OASL* genes. In this study, we first investigated the antiviral activity of goose OASL (goOASL) protein against a duck-origin Tembusu virus (DTMUV) in duck embryo fibroblast cells (DEFs). To investigate the relationship of conserved amino acids that are related to OAS enzyme activity and ubiquitin-like (UBL) domains with the antiviral activity of goOASL, a series of mutant goOASL plasmids was constructed, including goOASL-S64C/D76E/D78E/D144T, goOASL∆UBLs and goOASL∆UBLs-S64C/D76E/D78E/D144T. Interestingly, all these mutant proteins significantly inhibited the replication of DTMUV in DEFs in a dose-dependent manner. Immunofluorescence analysis showed that the goOASL, goOASL-S64C/D76E/D78E/D144T, goOASL∆UBLs and goOASL∆UBLs-S64C/D76E/D78E/D144T proteins were located not only in the cytoplasm where DTMUV replicates but also in the nucleus of DEFs. However, the goOASL and goOASL mutant proteins were mainly colocalized with DTMUV in the cytoplasm of infected cells. Our data indicated that goOASL could significantly inhibit DTMUV replication in vitro, while the active-site residues S64, D76, D78 and D144, which were associated with OAS enzyme activity, the UBL domains were not required for the antiviral activity of goOASL protein.

## 1. Introduction

The 2′-5′-oligoadenylate synthetase (OAS) family proteins are interferon (IFN)-induced antiviral factors and belong to the nucleotidyltransferase (NTase) superfamily [1,2]. There are four members in the OAS protein family: OAS1, OAS2, OAS3, 2′-5′-oligoadenylate synthetase-like (OASL) [3,4]. All of them have an NTase domain but contain one, two, three and one OAS units, respectively. However, there are normally two ubiquitin-like (UBL) domains located in the C-terminus of OASL protein, which is different from the other three OAS members [5]. Five conserved motifs have been identified in the OAS protein family: P-loop, D-box, LIRL, YALELLT and RPVILDPADP [6].

When activated by double-stranded RNA (dsRNA), the enzymatically active OAS protein can polymerize ATP into 2′, 5′-linked oligoadenylate (2-5A), depending on its nucleotidyltransferase (NTase) activity [7]. 2-5A is the activator of endoribonuclease L (RNase L), the activated RNase L can block viral replication by degrading viral and cellular single-stranded RNA [8]; thereby, the NTase activity plays an important role in the process of 2-5A production and is required for OAS enzyme activity. Some conserved residues associated with NTase activity were identified in some reports, including Gly-Ser in the P-loop motif and Asp in D-box [9,10]; these residues were obviously related to the OAS enzyme activity. There have been some reports that the mutation of two of three of the Asp residues in D-box can lead to loss of OAS protein enzyme activity, as identified in porcine OAS1 (pOAS1) and mouse OASL2 (mOASL2) [11,12]. However, not all OAS proteins have OAS enzyme activity, such as human OASL (huOASL), mouse OASL1 (mOASL1) and mouse Oas1b (mOAS1b) [4,13,14,15]. Other known OAS proteins that possess enzyme activity include human OAS1 (huOAS1), mOASL2, chicken OAS*A (chOAS*A), chicken OAS*B (chOAS*B) [4,14,16].

The antiviral effect of overexpressed RNase L against RNA virus was reported as early as in 1998 [17]. The antiviral function of RNase L against West Nile virus (WNV), which is a member of the genus *Flavivirus*, was reported in 2006 [18]. This antiviral mechanism of RNase L relied on the OAS protein and was known as the OAS/RNase L antiviral pathway. A novel antiviral pathway of enzymatically inactive OASL protein was recently identified. Overexpression of huOASL blocked the replication of multiple viruses in a RIG-I-dependent manner through interacting with RIG-I via the C-terminal UBL domains and enhanced RIG-I-mediated IFN induction [19,20]. This mechanism was named the OASL/RIG-I antiviral pathway. Therefore, it appears that both enzyme activity and UBL domains in the OAS family play important roles in antiviral function.

Several reports have shown that OAS proteins from humans, mice, pigs and chickens exhibit antiviral activity against flavivirus infection [21]. For example, hOAS1 (p42 and P46), human OAS3 (hOAS3) and human OASL (hOASL) can block the replication of type 2 dengue virus (DENV) in human cells [19,22]. Porcine OAS1 (pOAS1), porcine OAS2 (pOAS2) and porcine OASL (pOAS3) exhibit anti-Japanese encephalitis virus (JEV) activity in PK-15 cells [23]. MOAS1b and chOAS*A exhibit antiviral activity against WNV in mouse cells [24,25]. Only the OASL protein, which consisted of the N-terminal NTase domain, one OAS domain and two C-terminal UBL domains, was found in geese [26]. The conserved amino acids associated with OAS enzyme activity were observed in goose OASL (goOASL) [26]. Our previous research reported that as an IFN-induced antiviral protein, goOASL exhibited antiviral activity against Newcastle disease virus (NDV) in vitro, it was also involved in host anti-duck Tembusu virus (DTMUV) in vivo and in vitro [26,27]. DTMUV is a single-stranded positive-sense RNA virus, which was discovered in 2010, it belongs to the family *Flaviviridae* and the genus *Flavivirus* [28,29]. Both ducks and geese are susceptible to DTMUV [30,31]; the most common symptoms include a decline in egg production and nervous system disorders [32,33].

In this study, we investigated the antiviral function of goOASL against DTMUV in duck embryo fibroblast cells (DEFs). Moreover, a series of mutant plasmids with an OAS active-site mutation or UBL domain truncation for goOASL was constructed, their antiviral functions were explored in DEFs. The cellular colocalization of goOASL or mutant proteins with DTMUV were also investigated in DEFs. This study contributes to research on the antiviral mechanism of goOASL against DTMUV and explores the relationship between the antiviral effect and the OAS enzyme activity of goOASL.

## 2. Materials and Methods

### 2.1. Cells and Viruses

Embryonated duck eggs were obtained from the waterfowl breeding center of Sichuan Agricultural University (Ya’an city, Sichuan Province). Primary cells from 10-day-old duck embryos were prepared using standard dissociation procedures. The cells were cultured in Dulbecco’s modified Eagle’s medium (DMEM) (Gibco, Gaithersburg, MD, USA) supplemented with 10% fetal bovine serum (FBS) (Gibco, Gaithersburg, MD, USA), 100 IU/mL penicillin and 100 µg/mL streptomycin (Gibco, Gaithersburg, MD, USA) at 37 °C in a 5% CO_2_ incubator.

The duck-origin Tembusu virus (DTMUV) CQW1 strain (GenBank Accession: KM233707) was provided by the Research Center of Avian Diseases, Sichuan Agricultural University [34], the virus titer was 10^6^ TCID_50_/0.1 mL.

The animal studies were approved by the Institutional Animal Care and Use Committee of Sichuan Agricultural University (No. XF2014-18) and followed the National Institutes of Health guidelines for the performance of animal experiments.

### 2.2. RNA Extraction and cDNA Preparation

Total RNA from the cells was extracted using RNAiso plus reagent (TaKaRa, Dalian, China) according to the manufacturer’s instruction. cDNA was synthesized using 5× All-In-One RT Master Mix (Abm, Richmond, BC, Canada) according to the following program: 25 °C for 10 min, 42 °C for 15 min, 85 °C for 5 min. All cDNA samples were stored at −80 °C until used.

### 2.3. Western Blotting Assay

Protein samples added with 20% protein loading buffer (TransGen Biotech, Beijing, China) were boiled for 15 min, 20 µL samples were electrophoresed via SDS-PAGE and transferred onto polyvinylidene fluoride (PVDF) membranes (Bio-Rad, Hercules, CA, USA). After washing in Tris-buffered saline-Tween 20 (TBST) three times, the membranes were blocked at 37 °C in TBST with 5% skim milk for 1 h. After washing 3 times, the membranes were incubated with the primary antibodies mouse monoclonal anti-His antibody (Ruiyingbio, Suzhou, China) or mouse monoclonal anti-β-actin antibody (Ruiyingbio, Suzhou, China) diluted in TBST with 2.5% skim milk for 1 h at 37 °C. The secondary antibody HRP-goat anti-mouse IgG (Ruiyingbio, Suzhou, China) was incubated using the same method as the primary antibody. At the last step, the enhanced chemiluminescence (ECL) reagent (Bio-Rad, Hercules, CA, USA) was used for visualizing the bands, images were collected using the ChemiDoc MP imaging system (Bio-Rad, Hercules, CA, USA).

### 2.4. The Construction of goOASL-Mutant Plasmids

**Figure 1:**
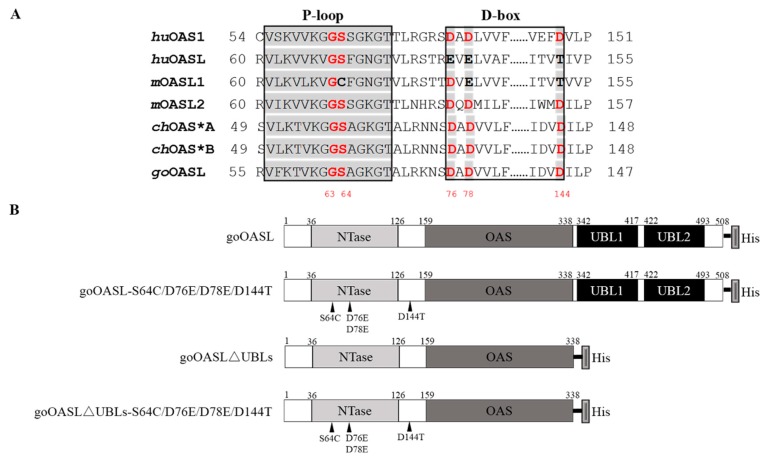
Construction of the eukaryotic expression plasmids of goOASL-mutant proteins. (**A**) Multiple sequence alignment of the P-loop and D-box amino acids of human OAS1 (huOAS1) (BAA00047.1), human OASL (huOASL) (AIC55448.1), mouse OASL1 (mOASL1) (AAM08092.1), mouse OASL2 (mOASL2) (NP-035984.2), chicken OAS*A (chOAS*A) (BAB19016.1), chicken OAS*B (chOAS*B) (NP-990372.1) and goose OASL (goOASL) (KU058695) proteins. The grey portion in the two boxes indicates conserved P-loop and D-box motifs, red indicates important amino acids identified in the NTase family; (**B**) Schematic diagram of the goOASL protein and its mutant proteins including goOASL-S64C/D76E/D78E/D144T, goOASL∆UBLs and goOASL∆UBLs-S64C/D76E/D78E/D144T. Each conserved NTase domain, OAS domain and UBL domain of the proteins and their corresponding amino acid position are marked; the black triangle indicates the mutation site.

As a member of the NTase superfamily, the conserved NTase active-site residues G62, S63, D75, D77 and D148 located in the P-loop (G62 and S63) and D-box (D75, D77 and D148) motifs of huOAS1 protein were found in goOASL protein (G63, S64, D76, D78 and D144) through multiple sequence alignment of huOAS1 (BAA00047.1), huOASL (AIC55448.1), mOASL1 (AAM08092.1), mOASL2 (NP_035984.2), chOAS*A (BAB19016.1), chOAS*B (NP_990372.1), goOASL (KU058695) proteins (Figure 1A) [9]. To investigate the relationship of the conserved residues of goOASL protein (G63, S64, D76, D78, D144 mapping in the goOASL protein sequence) with its antiviral activity against DTMUV, a mutant goOASL plasmid named goOASL-S64C/D76E/D78E/D144T was constructed in which amino acids associated with OAS activity were replaced with the corresponding amino acids on the P-loop and D-box motifs of enzymatically inactive huOASL and mOASL1 proteins (Figure 1B). The first 476 bp cDNA sequence in the N-terminus of goOASL-S64C/D76E/D78E/D144T was synthesized by the Beijing Genomics Institute; the remaining sequence was amplified via PCR using specific primers (listed in Table 1). Finally, a full-length sequence of goOASL-S64C/D76E/D78E/D144T was obtained via fusion PCR with a His-tag in a reverse primer (listed in Table 1). The full-length sequence was then subcloned into the pcDNA3.1 (+) expression vector using a ClonExpress^®^ II One Step Cloning Kit (Vazyme, Nanjing, China). To investigate the effect of C-terminal UBL domains in the antiviral activity of goOASL protein, a truncated mutant plasmid pcDNA3.1 (+)-goOASL∆UBLs named goOASL∆UBLs was constructed with specific primers (listed in Table 1) through a one-step cloning method (Figure 1B). A truncated mutant plasmid of goOASL∆UBLs with a S64C/D76E/D78E/D144T mutation, named goOASL∆UBLs-S64C/D76E/D78E/D144T, was constructed to further investigate the role of the S64, D76, D78, D144 residues and the UBL domains in goOASL antiviral activity (Figure 1B). In addition, to identify the importance of the two Asp residues in D-box, which is associated with OAS enzyme activity, in the goOASL antiviral activity, the mutant plasmid pcDNA3.1 (+)-goOASL-D76A/D78A, named goOASL-D76A/D78A, was further constructed with specific point mutation primers (listed in Table 1) (Figure S1A) in which the DADA mutation could cause the loss of OAS enzyme activity identified in mOASL2 protein [12]. Based on this strategy, another expression plasmid pcDNA3.1 (+)-goOASL∆UBLs-D76A/D78A, named goOASL∆UBLs-D76A/D78A, was constructed to investigate the importance of the D76 and D78 residues and the UBL domains in the antiviral activity of goOASL protein (Figure S1A). All expression plasmids were confirmed by sequencing.

### 2.5. The Cytotoxicity Assay of goOASL-Mutant Proteins

A cytotoxicity assay was performed to identify whether goOASL and its mutant proteins showed cytotoxicity. The DEFs seeded in a 96-well plate were transfected with goOASL, goOASL-S64C/D76E/D78E/D144T, goOASL∆UBLs and goOASL∆UBLs-S64C/D76E/D78E/D144T (0.1 µg/well) using TransIn EL Transfection Reagent (TransGen Biotech, Beijing, China). Cells transfected with the pcDNA3.1 (+) vector were used as the negative control. Untransfected cells were used as the blank control. At 24 h after transfection, the cells were treated with CCK-8 reagent (Beyotime, Shanghai, China) for 3 h, the optical density (OD) value of all samples was measured at 450 nm, the cell viability was calculated as follows: cell viability (%) = (OD of the experimental group − OD of the blank control)/(OD of the negative control − OD of the blank control) × 100.

### 2.6. Antiviral Activity Assay of goOASL and Its Mutant Proteins

DEFs seeded in a 12-well plate were transfected with goOASL (1.6 µg/well) for 24 h, 36 h and 48 h; the cell substrates were harvested and lysed with radio immunoprecipitation assay (RIPA) lysis buffer and stored for western blotting analysis. To detect the antiviral activity of goOASL protein against DTMUV, the DEFs were transfected with pcDNA3.1 (+) vector (negative control) and goOASL (1.6 µg/well) for 24 h. Later, the cells were infected with DTMUV (10^4^ TCID_50_/well) and collected at 24 h and 36 h after infection with RNAiso plus reagent.

DEFs seeded in a 12-well plate were transfected with pcDNA3.1 (+) vector, goOASL, goOASL-S64C/D76E/D78E/D144T, goOASL∆UBLs and goOASL∆UBLs-S64C/D76E/D78E/D144T (1.6 µg/well). At 24 h after transfection, the cell substrates were collected with RIPA buffer and used for western blotting analysis. To investigate the antiviral activity of goOASL-mutant proteins, the DEFs were transfected with pcDNA3.1 (+) vector, goOASL, goOASL-S64C/D76E/D78E/D144T, goOASL∆UBLs and goOASL∆UBLs-S64C/D76E/D78E/D144T, respectively, for 24 h. Later, the cells were infected with DTMUV (10^4^ TCID_50_/well), the cell substrates were collected at 24 h. Furthermore, the cells were transfected with these plasmids at different doses from 1.6 µg/well to 0.8 µg/well and 0.4 µg/well. At 24 h after transfection, the cells were infected with DTMUV (10^5^ TCID_50_/well) and harvested at 24 h. In addition, the cells were also transfected with goOASL-D76A/D78A and goOASL∆UBLs-D76A/D78A, respectively. At 24 h after transfection, the goOASL-D76A/D78A-overexpressing cells, goOASL∆UBLs-D76A/D78A-overexpressing cells and control cells were infected with DTMUV (10^4^ TCID_50_/well) and collected at 24 h after infection.

The genomic copy number of DTMUV was detected via qRT-PCR using the EvaGreen 2× qPCR MasterMix (Abm, Richmond, BC, Canada) and a Real-Time Detection System (Bio-Rad CFX96, Hercules, CA, USA) with the following program: 95 °C for 10 min, followed by 39 cycles of 95 °C for 15 s, 56 °C for 1 min. The absolute quantitative standard curve for DTMUV was built based on the pMD19-T-DTMUV-E plasmid, the targeted product was 224 bp. The specific primers for DTMUV detection are listed in Table 1.

### 2.7. The Cellular Colocation of goOASL and Its Mutant Proteins with DTMUV

DEFs were seeded on 20-mm glass slides in a 12-well tissue culture plate. When the cell monolayer reached 50% confluence, the cells were transfected with pcDNA3.1 (+) vector, goOASL, goOASL-S64C/D76E/D78E/D144T, goOASL∆UBLs, or goOASL∆UBLs-S64C/D76E/D78E/D144T. At 24 h after transfection, the infected group was infected with DTMUV (10^4^ TCID_50_/well). After 12 h, both the infected group and the uninfected group were washed with 1× phosphate-buffered saline (PBS) and fixed with 4% paraformaldehyde (0.5 mL/well) diluted with 1× PBS overnight at 4 °C. Later, the cells were washed with 1× PBS-Tween 20 (PBST) three times and treated with 0.3% Triton X-100 (0.5 mL/well) to react for 1 h at 4 °C. Next, the cells were washed with 1× PBST and blocked with 3% bovine serum albumin-PBS (BSA-PBS) (0.5 mL/well) for 1 h at 37 °C. The cells were washed with 1× PBST and incubated with rabbit monoclonal anti-His antibody (Cell Signaling Technology, Shanghai, China) diluted 400 times and mouse polyclonal anti-DTMUV antibody diluted 200 times (the primary antibody, 200 µL/well) for 1.5 h at 37 °C. Next, cells were incubated with tetraethyl rhodamine isothiocyanate (TRITC)-goat anti-rabbit IgG antibody (Thermo, Waltham, MA, USA) or fluorescein isothiocyanate (FITC)-goat anti-mouse IgG antibody (Thermo, Waltham, MA, USA) (secondary antibody diluted to 200 times, 200 µL/well) for 1 h at 37 °C in the dark. Finally, the cells were incubated with 4′, 6-diamidino-2-phenylindole (DAPI) (250 µL/well) diluted 150 times for 15 min at 37 °C in the dark. Red, blue and green fluorescence was detected via fluorescence microscopy (Nikon Eclipse 80i, Tokyo, Japan) (magnification 600×) and analysed using Image Pro Plus 6.0 (Media Cybernetics, Rockville, MD, USA).

## 3. Results

### 3.1. The Antiviral Activity of goOASL Protein against DTMUV in DEFs

**Figure 2:**
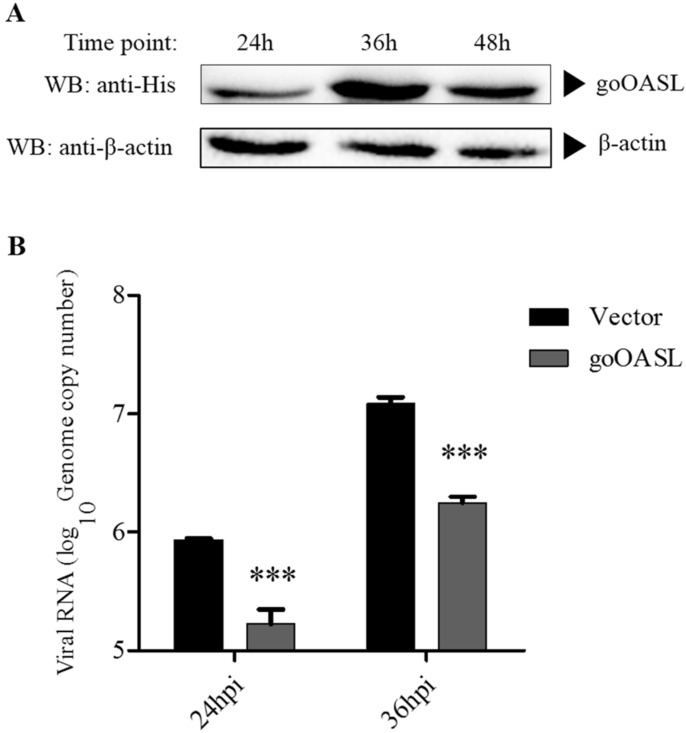
The antiviral activity of goose 2′-5′-oligoadenylate synthetase-like (goOASL) protein against duck-origin Tembusu virus (DTMUV) in duck embryo fibroblast cells (DEFs). (**A**) Overexpression of goOASL in DEFs. DEFs seeded in a 12-well plate were transfected with goOASL (1.6 µg/well), the cell substrates were harvested using RIPA buffer at 24 h, 36 h and 48 h after transfection. The cell lysates were collected for western blotting analysis. Mouse monoclonal anti-His antibody or mouse monoclonal anti-β-actin antibody was used as the primary antibody, HRP-goat anti-mouse IgG was used as the secondary antibody; (**B**) Overexpression of goOASL protein inhibited the replication of DTMUV in DEFs. GoOASL-overexpressing DEFs and control cells transfected with pcDNA3.1 (+) vector were infected with DTMUV (10^4^ TCID_50_/well) at 24 h after transfection. At 24 h and 36 h, the genome copy number of DTMUV in the cells was quantified by qRT-PCR. All data were analysed using GraphPad Prism software and were represented as the means ± SD (*n* = 3). The significance was determined with the unpaired two-tailed *t*-test (*** *p* < 0.001).

Western blotting analysis showed that the goOASL protein could be detected at 24 h, 36 h and 48 h in DEFs transfected with goOASL (Figure 2A). The protein levels of goOASL at 36 h and 48 h were higher than that at 24 h. The antiviral experiment showed that the genome copy number of DTMUV in the goOASL-overexpressing cells significantly declined compared with the control cells at 24 h and 36 h (Figure 2B).

### 3.2. The Antiviral Activity of goOASL-Mutant Proteins against DTMUV in DEFs

**Figure 3:**
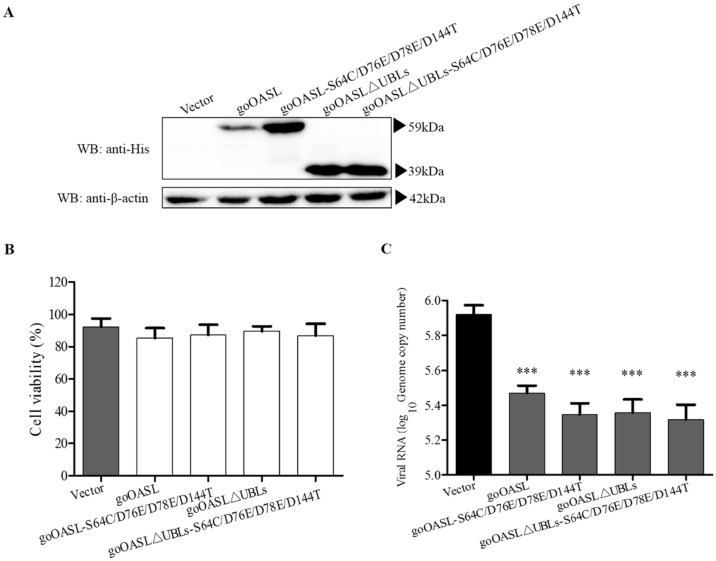
The antiviral activity of goOASL and goOASL-mutant proteins against DTMUV in DEFs. (**A**) Overexpression of goOASL-mutant proteins in DEFs. DEFs seeded in a 12-well plate were transfected with pcDNA3.1 (+) vector, goOASL, goOASL-S64C/D76E/D78E/D144T, goOASL∆UBLs and goOASL∆UBLs-S64C/D76E/D78E/D144T (1.6 µg/well), respectively. At 24 h after transfection, the cells were harvested using RIPA buffer for western blotting analysis. Mouse monoclonal anti-His antibody or mouse monoclonal anti-β-actin antibody was used as the primary antibody, HRP-goat anti-mouse IgG was used as the secondary antibody; (**B**) Detection of the cytotoxicity of goOASL and its mutant proteins in DEFs. The optical density (OD) value of all samples was measured at 450 nm after the goOASL-, goOASL-S64C/D76E/D78E/D144T-, goOASL∆UBLs- and goOASL∆UBLs-S64C/D76E/D78E/D144T-overexpressed cells and control cells were treated with CCK-8 reagent for 3 h. Cell viability was calculated using the standard formula; (**C**) GoOASL-mutant proteins exhibited antiviral activity against DTMUV in DEFs. At 24 h after transfection, the goOASL-, goOASL-S64C/D76E/D78E/D144T-, goOASL∆UBLs- and goOASL∆UBLs-S64C/D76E/D78E/D144T-overexpressed cells and control cells were infected with DTMUV at 24 h after transfection with the pcDNA3.1 (+) vector (10^4^ TCID_50_/well). At 24 h, the genome copy number of DTMUV in the cells was quantified via qRT-PCR. All data were analysed using GraphPad Prism software and were represented as the means ± SD (*n* = 3). Significance was determined via the unpaired two-tailed *t*-test (*** *p* < 0.001).

Western blotting assay showed that the goOASL-mutant proteins goOASL-S64C/D76E/D78E/D144T, goOASL∆UBLs, goOASL∆UBLs-S64C/D76E/D78E/D144T could be successfully expressed in DEFs at 24 h after transfection (Figure 3A). The recombinant goOASL-S64C/D76E/D78E/D144T, goOASL∆UBLs and goOASL∆UBLs-S64C/D76E/D78E/D144T proteins were approximately 59 kDa, 39 kDa and 39 kDa, respectively. The cytotoxicity assay showed that the goOASL protein and its mutant proteins exhibited no cytotoxicity in transfected DEFs (Figure 3B). The antiviral activity experiment showed that goOASL-S64C/D76E/D78E/D144T, goOASL∆UBLs and goOASL∆UBLs-S64C/D76E/D78E/D144T significantly inhibited the replication of DTMUV, similar to the goOASL protein, at 24 h in DEFs (Figure 3C). Western blotting analysis showed that both goOASL-D76A/D78A and goOASL∆UBLs-D76A/D78A proteins could be successfully expressed in DEFs (Figure S1B), they did not induce toxicity to DEFs according to the cytotoxicity assay (Figure S1C). qRT-PCR showed that the genome copy number of DTMUV in the goOASL-D76A/D78A- and goOASL∆UBLs-D76A/D78A-overexpressed DEFs significantly decreased compared with that in the control cells (Figure S1D).

### 3.3. GoOASL and Its Mutant Proteins Exhibited Antiviral Activity against DTMUV in DEFs in A Dose-Dependent Manner

**Figure 4:**
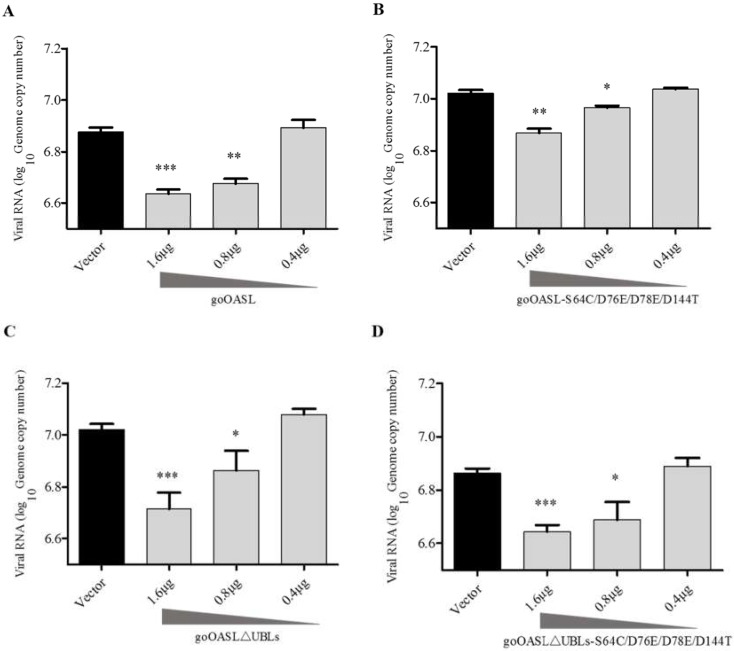
GoOASL and its mutant proteins exhibited antiviral activity against DTMUV in DEFs in a dose-dependent manner. GoOASL-overexpressing cells (**A**); goOASL-S64C/D76E/D78E/D144T-overexpressing cells (**B**); goOASL∆UBLs-overexpressing cells (**C**); goOASL∆UBLs-S64C/D76E/D78E/D144T-overexpressing cells (**D**) were transfected with different doses of plasmids, including 1.6 µg/well, 0.8 µg/well, 0.4 µg/well; the control cells were transfected with pcDNA3.1 (+) vector (1.6 µg/well); all these cells were infected with DTMUV (10^5^ TCID_50_/well) at 24 h after transfection. After 24 h, the genome copy number of DTMUV in the cells was quantified via qRT-PCR. All data were analysed via GraphPad Prism software and were represented as the means ± SD (*n* = 3). Significance was determined via the unpaired two-tailed *t*-test (* *p* < 0.05; ** *p* < 0.01; *** *p* < 0.001).

The goOASL protein and its mutant proteins did not exhibit an antiviral effect against DTMUV in DEFs at a low transfection dose (0.4 µg/well), while all of them significantly inhibited DTMUV replication in DEFs at higher transfection doses (1.6 µg/well and 0.8 µg/well) (Figure 4A–D). In general, the goOASL protein and its mutant proteins showed antiviral activity against DTMUV in DEFs in a dose-dependent manner.

### 3.4. Cellular Colocalization of goOASL and Its Mutant Proteins with DTMUV in DEFs

**Figure 5:**
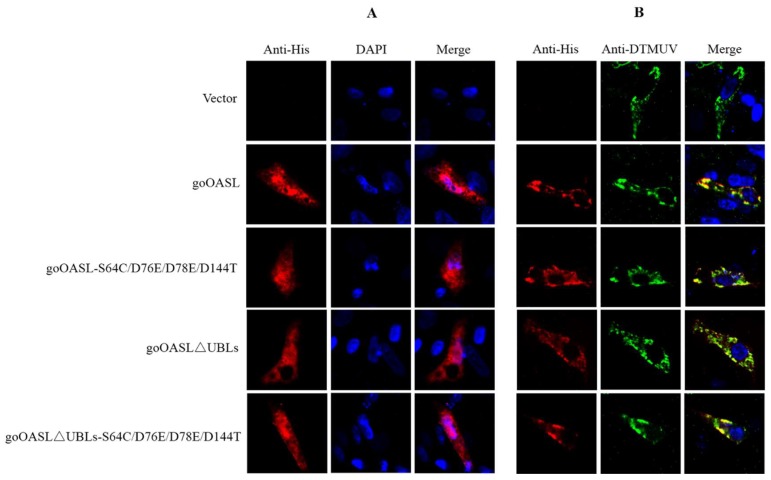
Cellular localization of goOASL and its mutant proteins in DEFs. (**A**) Cellular localization of goOASL and its mutant proteins in normal DEFs. DEFs seeded on 20-mm glass slides in a 12-well tissue culture plate were transfected with goOASL, goOASL-S64C/D76E/D78E/D144T, goOASL∆UBLs, or goOASL∆UBLs-S64C/D76E/D78E/D144T (1.6 µg/well). Immunofluorescence was detected at 36 h after transfection via fluorescence microscopy; (**B**) Cellular colocalization of goOASL and its mutant proteins in DEFs with DTMUV. DEFs seeded on the 20-mm glass slides in the 12-well tissue culture plate were transfected with goOASL, goOASL-S64C/D76E/D78E/D144T, goOASL∆UBLs, or goOASL∆UBLs-S64C/D76E/D78E/D144T (1.6 µg/well). At 24 h after transfection, the cells were infected with DTMUV (10^4^ TCID_50_/well) for another 12 h. Immunofluorescence was detected using fluorescence microscopy. The rabbit anti-His antibody and mouse anti-DTMUV antibody were used as primary antibodies, the TRITC-goat anti-rabbit IgG and FITC-goat anti-mouse IgG were used as secondary antibodies. DAPI was used for nucleolus staining. Fluorescence (red, green and blue) was detected via fluorescence microscopy (magnification 600×) and analysed using Image Pro Plus 6.0.

Indirect immunofluorescent assay was conducted to explore the cellular localization of goOASL, goOASL-S64C/D76E/D78E/D144T, goOASL∆UBLs, goOASL∆UBLs-S64C/D76E/D78E/D144T proteins in DEFs with or without DTMUV infection. The data showed that the goOASL, goOASL-S64C/D76E/D78E/D144T, goOASL∆UBLs, goOASL∆UBLs-S64C/D76E/D78E/D144T proteins were evenly distributed in both the cytoplasm and nucleus at 36 h in the uninfected group, while in the infected group, goOASL and its mutant proteins were mainly colocalized with DTMUV in the cytoplasm (Figure 5).

## 4. Discussion

The *OASL* gene has been identified in almost all birds, while the *OAS1*, *OAS2* and *OAS3* genes are not found in any birds except ostrich, which has both the *OASL* gene and the *OAS1* gene [35]. The goOASL protein has two additional UBL domains compared with the OAS1, OAS2 and OAS3 proteins, similar to huOASL protein. An article published in 2014 indicated that overexpression of huOASL protein inhibited the replication of some viruses through interacting with RIG-I and enhanced RIG-I-mediated IFN induction [19,20]. A recent article published in 2017 reported that overexpressed porcine OASL (pOASL) protein suppressed the replication of classical swine fever virus (CSFV) through interacting with MDA5 and activated the MDA5-mediated type I IFN pathway [36]. However, the antiviral mechanism of goOASL is still unknown.

Our antiviral assay in DEFs showed that goOASL protein exhibited antiviral activity against DTMUV; a similar finding has been reported recently for duck OASL protein, which showed an antiviral effect against TMUV in DF-1 cells [37]. From the experimental results, we observed that the mutations in the conserved active-site amino acids of the NTase family did not influence the antiviral effect of goOASL protein. This result suggested that the key amino acids for NTase activity were not required for the antiviral activity of goOASL protein, similar to enzymatically inactive huOASL protein, which showed no NTase activity but exhibited antiviral activity against hepatitis C virus (HCV) [38]. This result also suggested that the conserved amino acids in D-box associated with the OAS enzyme activity were not required for the antiviral activity of goOASL protein (Figure S1D). Furthermore, mOASL2 and chOAS*A could inhibit virus replication even though two of the three Asp residues of D-box were substituted with Ala or three Asp residues of D-box were deleted [12,25]. We assumed that the antiviral effect of goOASL protein against DTMUV might be independent of OAS enzyme activity because the mutation of the key amino acids associated with OAS enzyme activity did not influence its antiviral function. The data also showed that UBL domain truncation did not impact the antiviral effect of goOASL protein against DTMUV in DEFs. The data suggested that two UBL domains of the goOASL protein were not required for its antiviral effect. This characteristic of the goOASL protein is similar to the chOAS*A protein: when UBL1 and UBL2 of chOAS*A protein are deleted, chOAS*A∆UBL1/UBL2 protein can still suppress WNV replicon replication [25]. This result is also similar to pOASL protein, which naturally lack the C-terminal UBL domains but still exhibit an antiviral effect against JEV [23]. It appeared that the two C-terminal UBL domains of the OASL protein were not important for their antiviral function, except for the huOASL protein [12]. Collectively, our data indicated that both active-site residues associated with OAS activity and C-terminal UBL domains were unnecessary for the antiviral effect of goOASL protein against DTMUV in vitro.

Immunofluorescence analysis further showed that the goOASL, goOASL-S64C/D76E/D78E/D144T, goOASL∆UBLs, goOASL∆UBLs-S64C/D76E/D78E/D144T proteins were evenly located not only in the cytoplasm but also in the nucleus. Unexpectedly, during DTMUV infection, goOASL and its mutant proteins were all located in the cytoplasm and were codistributed with DTMUV. DTMUV is a member of the family *Flaviviridae*, genus *flavivirus*. In flavivirus-infected cells, the virus enters cells through receptor-mediated endocytosis; the nucleocapsid is released into the cytoplasm, both viral RNA synthesis and virus assembly occur in the cytoplasm in association with the endoplasmic reticulum (ER) membrane [39,40,41]. Our data suggested that the antiviral effect of goOASL protein against DTMUV might occur in the cytoplasm. A previous report indicated that the NS5A protein of HCV, a member of the family *Flaviviridae*, could physically interact with hOAS1 (p40) protein and mouse OAS1 (p42) protein and could interfere with 2-5AS functions [42]. Further research is needed to determine whether the same interaction exists between goOASL protein and DTMUV NS5 protein.

In summary, we preliminarily explored the relationship between element amino acids or UBL domains and the antiviral effect of goOASL protein against DTMUV in DEFs. The currently known antiviral mechanisms of OAS proteins involve enzymatically active huOAS1 protein and pOAS1 protein, which inhibit DENV-2 and JEV replication in an RNase L-dependent manner. Enzymatically inactive huOASL protein suppresses the replication of multiple viruses including DENV-2 in a RIG-I-dependent manner, pOASL protein, which lacks C-terminal UBL domains, congenitally inhibits CSFV in a MDA5-dependent manner. Additionally, the mechanism underlying the antiviral action of mOAS1b protein and chOAS*A protein remains unclear; both effects appear to be RNase L-independent and RIG-I-independent. Here, we speculated that goOASL protein inhibited the replication of DTMUV in an RNase L-independent manner because goOASL protein with mutations of key residues related to OAS activity still exhibited antiviral activity against DTMUV. It also appeared that goOASL protein did not inhibit the replication of DTMUV in a RIG-I-dependent manner because goOASL protein with truncated UBLs still exhibited antiviral activity. However, the antiviral mechanism of goOASL protein needs to be uncovered in future work.

## Figures and Tables

**Table 1:** List of Primers.

Primers	Sequence (5′-3′)	Application
goOASL-N1-F goOASL-N1-R	CTGCGGGAGCCGCGATGGAG GGGCATCGTAGGTGGGCAGGA	Amplification of the N-terminus of goOASL-S64C/D76E/D78E/D144T
goOASL-C2-F goOASL-C2-R	TCCTGCCCACCTACGATGCCC CCAGGGAGAAATAAAAGGGGATG	Amplification of the rest of the sequence of goOASL-S64C/D76E/D78E/D144T
goOASL-His-F goOASL-His-R	TGGTGGAATTCTGCAGATATCGCCACCATGGAGCTGCGGGACGTG GCCCTCTAGACTCGAGCGGCCGCTCAGTGGTGGTGGTGGTGGTGGGAGGGCTGGCAGCAAGG	Amplification of goOASL-S64C/D76E/D78E/D144T and goOASL-D76A/D78A
goOASL∆UBLs-His-F goOASL∆UBLs-His-R	TGGTGGAATTCTGCAGATATCGCCACCATGGAGCTGCGGGACGTG GCCCTCTAGACTCGAGCGGCCGCTCAGTGGTGGTGGTGGTGGTGCGGCTGCACGTTCCAGGGGT	Amplification of goOASL∆UBLs, goOASL∆UBLs-S64C/D76E/D78E/D144T and goOASL∆UBLs-D76A/D78A
goOASL-N1′-F goOASL-N1′-R	CTGCGGGAGCCGCGATGGAG CCACGGCGGCGGCCGAGTTCT	Amplification of the N-terminus of goOASL-D76A/D78A
goOASL-C2′-F goOASL-C2′-R	AGAACTCGGCCGCCGCCGTGG CCAGGGAGAAATAAAAGGGGATG	Amplification of the rest of the sequence of goOASL-D76A/D78A
DTMUV(E)-F DTMUV(E)-R	CGCTGAGATGGAGGATTATGG ACTGATTGTTTGGTGGCGTG	qRT-PCR for detection of viral RNA of DTMUV

## Supplementary Materials

Supplementary materials can be found at http://www.mdpi.com/1999-4915/10/7/371/s1.
