# Supplementary material for: Conserved Active-Site Residues Associated with OAS Enzyme Activity and Ubiquitin-Like Domains Are Not Required for the Antiviral Activity of goOASL Protein against Avian Tembusu Virus
Source: Viruses. 2018 Jul 15;10(7):371. doi: 10.3390/v10070371 (PMC6071104; doi:10.3390/v10070371)
Supplement: Supplementary file 1 [file viruses-10-00371-s001.zip › viruses-306032-SI.pdf]

Supplementary Material:

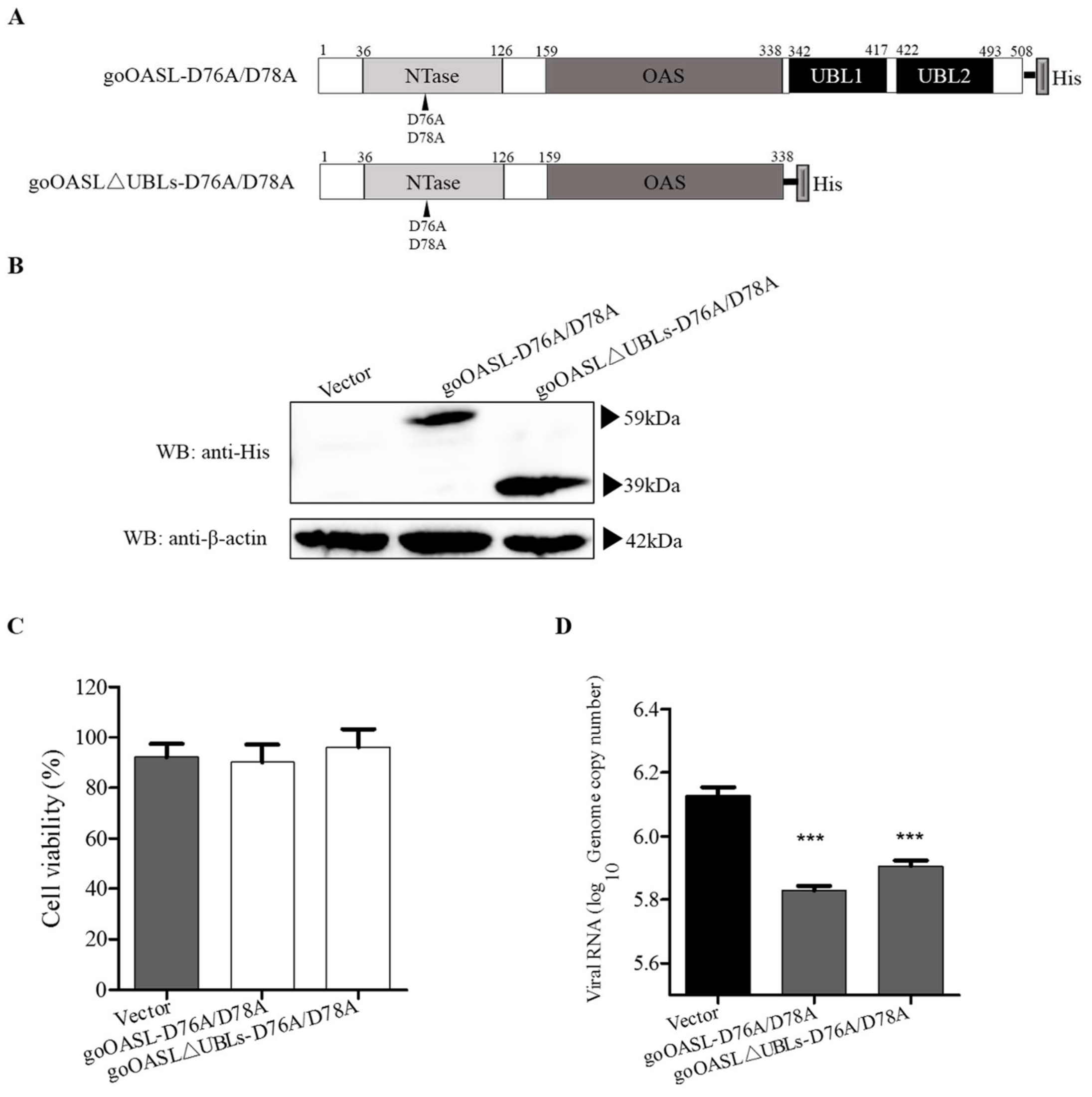

Figure S1. Construction of the eukaryotic expression plasmids of goOASL-mutant proteins and its antiviral activity against DTMUV in DEFs. (A) Schematic diagram of goOASL-D76A/D78A and goOASL▵UBLs-D76A/D78A. (B) Overexpression of goOASL-D76A/D78A and goOASL▵UBLs-D76A/D78A proteins in DEFs. DEFs seeded in a 12-well plate were transfected with goOASL-D76A/D78A and goOASL▵UBLs-D76A/D78A (1.6 μg/well), respectively. At 24 h after transfection, the cells were harvested using RIPA buffer for western blotting analysis. (C) Detection of cytotoxicity of the goOASL-D76A/D78A and goOASL▵UBLs-D76A/D78A proteins in DEFs (D) The overexpression of the goOASL-D76A/D78A and goOASL▵UBLs-D76A/D78A proteins inhibited the replication of DTMUV in DEFs. The

goOASL-D76A/D78A-overexpressing cells, goOASL∆UBLs-D76A/D78A-overexpressing cells and control cells were infected with DTMUV (104 TCID50/well) at 24 h after transfection. At 24 h, the genome copy number of DTMUV was quantified via qRT-PCR. All data were analysed using the GraphPad Prism software and were represented as the means + SD (n = 3). Significance was determined using the unpaired two-tailed t-test (***$p<0.001$).
